# A Case of Hemorrhagic Malarial Fever

**Published:** 1882-11

**Authors:** R. O. Engram

**Affiliations:** Montezuma, Ga.


### A CASE OF HEMORRHAGIC MALARIAL FEVER.

By R. O. ENGRAM, M.D., Montezuma, Ga.

Reported by Request.

E. W. W., white, aged twenty-one years, complexion dark. Was called June 18th, 1882, and found patient suffering from an attack of remittent fever, which he had experienced several times previously. He had considerable splenic enlargement at the time. In four or five days he was up and discharged, with directions to take a tonic of strychnia, iron and bark, which, however, he failed to follow. Again, on July 4th, was called to him in a similar attack. He was again up in a few days, and, as before, was instructed to take the same tonic, but, as on the former occasion, he failed to do so.

On August 21st I was called in haste to see him, and found him suffering from an attack of hemorrhagic malarial fever, with the following history: Considerable heat of the skin, which had been ushered in with a chill previous to my arrival; skin tinged a bright saffron color; conjuctiva yellow; nausea, with vomiting of viscid green bile; tongue moist, pointed, nervous, and coated with a dirty, yellowish fur; patient excited and nervous, with cephalic pain; the urine was beyond a deep port wine color, more of a dark red—what is termed black bloody urine; pulse 120; compressible.

In the morning, patient had ridden over to see my friend Dr. Watts, who, fearing the results, prescribed decided doses of gallic acid.

On arrival I gave twenty grains of gallic acid. The nausea was met with one-fourth of a grain of morphia, hypodermically, which acted well. I ordered quinine and salicylic acid, three grains each, every four hours; also gave a potion of sweet spirits of nitre and nitrate of potash every four hours as a diuretic, and directed an embrocation composed of equal parts of chloroform, spirits of turpentine and sweet oil to be applied over the region of the kidneys frequently. My friend, Dr. Watts, kindly saw the patient during the night, and found it necessary to administer morphia but once, as the stomach did well.

August 22d, I saw patient at nine o'clock A.M. with Dr. Watts; found him quiet, after a good night's rest; temperature, 101; pulse, 115; respiration, 21; specific gravity of urine, 1006; color of urine, slightly lighter; tongue not changed; skin moist and deeper saffron or orange hue; less jactition and nervousness than on the day before. Three o'clock P.M.: Not much change, except skin clearer; urine little lighter; no nausea; pulse same; temperature one-half of a degree less, and respiration 20. Left off salicylic acid and added paregoric to the diaphoretic mixture.

August 23d, nine o'clock A.M.: Dr. J. C. Hooks was called in during the night. Saw patient with Drs. Watts and Hooks, and found that he had passed through a very good night; pulse 90; temperature 99½; respiration 19; specific gravity of the urine 1008, clearing; tongue moist; skin lighter in color, and moist; patient apparently improving. Treatment continued, except the embrocation of oil, turpentine and chloroform. Turpentine stupes were substituted to be applied over the region of the kidneys every six hours.

Three o'clock P.M.: Patient doing well, except occasional nausea, for which we used pepsine, five grains, with a like quantity of Dover's powder, as required, upon which he did very well during the night.

August 24th, nine o'clock A.M.: Patient spent a tolerably fair night, growing restless towards morning. Pulse 129; temperature 102; respiration 24; specific gravity of the urine 1009; medium ley color, copious brown deposit on cooling; tongue dry, with dark, brownish-yellow coat; skin hot and dry; urine on being tested for albumen, both yesterday and to-day, showed about 20 per cent. of coagula; patient's condition serious. Foot baths and brandy were used quite freely, under which he showed some improvement in pulse and skin.

Three o'clock P.M.: Pulse 152; temperature 103; respiration 32; skin hot and dry, with a deep yellow color; nausea and vomiting quite frequent; great restlessness and jactitation; patient occasionally delirious, intermitting with listless indifference; condition quite critical. Under unstinted use of brandy and frequent pediluvia, reaction took place; the pulse improved, falling to 140 and 135; respiration reduced to 24; less jactation, and the night was passed with some rest.

August 25th: [The record for this day was accidentally omitted by the printer, and the manuscript was destroyed before the error was discovered.]

August 26th, nine o'clock A.M.: Pulse 98; temperature 99; respiration 17; urine clearing; specific gravity 1012; tongue moist; skin moist and pleasant. Same treatment continued.

August 27th: Patient improving; symptoms all better; urine quite clear; skin clearing rapidly; specific gravity of urine 1015. Saw patient no more, but he went on to recovery, under the attention of Dr. Watts.

Well, what do we notice unusual about this case? Nothing at variance with many other cases of the same disease; yet there are some things that are very peculiar about all of these cases, and among them I will enumerate the following conditions:

1. A large per cent. of albumen, with a low specific gravity—1006.

2. An abundant deposit of sediment, with a like low specific gravity—1008.

3. Grave symptoms presenting themselves just as the urine and skin seem to indicate speedy recovery and prompt return to health.

4. Improvement of pulse and other symptoms under alcoholic stimulants and hot foot baths.

I say we have abundant deposits, with low specific gravity. What is this deposit? Not the chlorides; they are almost wholly absent. We took the darkest, reddest looking specimen and hunted for blood corpuscles, but we could not get even the suspicious trace of one. We do not say blood corpuscles are not there, but that we could not find them. However, in the disease, under the field of the microscope, we found a free deposit of mixed urates, which should give us a high instead of low specific gravity. Now, it does not seem that we would have so low a specific gravity as 1006, which we found with this free deposit of urates.

Then, again, we found a specific gravity of 1009 on the 24th, at which time we submitted a specimen to the microscope, which brought out the following condition, which should certainly give a high specific gravity. This specimen showed long cylindrical bodies, tubular casts, oblong ovate bodies, epithelial cells from kidneys, round granular drops supposed to be pus corpuscles, stellate bodies and scattering urates, also a free deposit of nucleated epithelial cells and tubular casts from the kidneys, with here and there thickly-scattered bodies resembling in every way pus globules; also stellate bodies resembling urates, which seem to have almost disappeared since the first specimen was examined; yet with all these deposits and a copious coagula of albumen, we have a low specific gravity—1009. And just here we have to appear alarming symptoms. Can these deposits—viz.: pus globules, tubular casts and epithelial cells—tell of the terrible strain on the kidneys which have been conducting off the sewage of the body? And further, do these alarming symptoms, later in the day, indicate that the kidneys are being worked to destruction to carry off these deposits, which are manifoldly increased towards night? If so, how does brandy and the hot foot bath give relief? Is it by stimulating the kidneys to increased strength to bridge over the crisis of overwork, or by some direct sedative action upon the general system? We further find, as health returns, the urates increase in the urine and the specific gravity increases; and as the gravity of the disease increases, we have less urates and a lower specific gravity. What becomes of them? Are their elementary principles not caught up from the blood at the proper places, or are they not formed, but their elements retained in the blood? Or are they formed, but re-absorbed, and when the kidneys fail to throw them off, do they produce uræmic poisoning? If either or both of these propositions are true, then where does the fault lie? In a diseased liver, which is incapacitated from elaborating the blood and breaking it up into healthy complex bodies, or is it in diseased kidneys which fail to carry off the effete debris of the system? These and other questions are those which must be seriously considered in making up the therapeutics of this hitherto alarmingly fatal disease.

